# PB.50: Axillary lymph node ultrasound in breast cancer patients: what minimum threshold for diffuse cortical thickening predicts nodal involvement?

**DOI:** 10.1186/bcr3550

**Published:** 2013-11-08

**Authors:** L Clarke, A Leaver, P Newton

**Affiliations:** 1 The Queen Elizabeth Hospital, Gateshead, UK

## Introduction

In our Trust we classify axillary ultrasound findings as LN1 to LN5, performing fine needle aspiration cytology (FNAC) on LN3 to LN5, where LN3 represents diffuse cortical thickening (DCT) of greater than 2 mm. The resulting FNAC triages patients to either sentinel lymph node biopsy or axillary node dissection. The aim is that patients will undergo only one axillary surgical procedure. There is variation in the literature and between breast units in the DCT threshold for performing FNA, and unnecessary FNAs should be avoided. Does the resulting cytology and surgical histology validate our 2 mm threshold; or can the threshold be safely increased to 2.3 mm or 3 mm as used by some centres?

## Methods

The MDT records and images for all our invasive breast cancer patients classified axillary LN3 and operated upon in 2012 were reviewed. The positive predictive value (PPV) was calculated for ranges of DCT for a post-test probability of a C5 result at FNA, and then for the yield of 2+ positive nodes at surgery.

## Results

A total of 112 female patients were LN3 and underwent FNA in 2012. The PPV for a C5 result in DCT in ranges 2.0 to 2.29, 2.30 to 2.99 and ≥3.0 was 9.1% (1/11), 11.3% (6/53) and 14.6% (7/48) respectively. The PPV for a yield of 2+ malignant nodes was 9.1% (1/11), 9.4% (5/53) and 4.2% (2/48) respectively.

## Conclusion

These findings have validated our use of the 2 mm threshold for FNAC. Increasing the threshold would result in a significant number of women requiring a second axillary surgical procedure as current treatment guidelines stand.

